# Mutual Relations between Texture and Aroma of Cooked Rice—A Pilot Study

**DOI:** 10.3390/foods11223738

**Published:** 2022-11-21

**Authors:** Zihan Wang, Jun Wang, Xu Chen, Enpeng Li, Songnan Li, Cheng Li

**Affiliations:** 1 Jiangsu Key Laboratory of Crop Genomics and Molecular Breeding/Key Laboratory of Plant Functional Genomics of the Ministry of Education/Jiangsu Key Laboratory of Crop Genetics and Physiology, Agricultural College of Yangzhou University, Yangzhou 225009, China; 2 School of Health Science and Engineering, University of Shanghai for Science and Technology, Shanghai 200093, China; 3 School of Tourism and Cuisine, Yangzhou University, Yangzhou 225127, China; 4 Engineering Research Center of Health Food Design & Nutrition Regulation, Dongguan Key Laboratory of Typical Food Precision Design, China National Light Industry Key Laboratory of Healthy Food Development and Nutrition Regulation, School of Life and Health Technology, Dongguan University of Technology, Dongguan 523808, China; 5 Jiangsu Co-Innovation Center for Modern Production Technology of Grain Crops, Yangzhou University, Yangzhou 225009, China; 6 Joint International Research Laboratory of Agriculture and Agri-Product Safety of the Ministry of Education of China, Institutes of Agricultural Science and Technology Development, Yangzhou University, Yangzhou 225009, China

**Keywords:** hardness, stickiness, GC-IMS, rice volatiles, Pearson correlation

## Abstract

Texture and aroma are two important attributes for the eating quality of cooked rice, but their mutual relations are not clear. Cooked rice with a desirable texture might suffer from a deteriorated aroma property. To better understand the relations between texture and aroma, six different rice varieties with desirable eating qualities have been selected, with their texture and aroma profile characterized by a texture analyzer and gas chromatography-ion mobility spectrometry, respectively. A large variance of textural attributes and a total number of 39 major volatile organic components were observed for these cooked rice varieties. Pearson correlation showed that the hardness of cooked rice was positively correlated with the content of E-2-hexenal, 2-hexanol-monomer, 1-propanol, and E-2-pentenal, while stickiness was positively correlated with 5-methyl-2-furanmethanol and dimethyl trisulfide. Possible underneath mechanisms were discussed for these relations. These results could help the rice industry to develop rice products with both desirable texture and aroma property.

## 1. Introduction

Rice is one of the most important staple foods around the world for human consumption. More than 90% of rice is consumed in East, South, and Southeast Asia, where about 60% of the global population lives. Over the past thirty years, the demand for rice with higher quality is rapidly increasing with the high levels of economic growth, especially in Asia, lifting hundreds of millions of people out of poverty to the middle class. Eating quality is a critical factor in determining customer acceptance of a particular rice variety, and most consumers would not eat the cooked rice they dislike even though they are aware of the potential health benefits. For example, a survey of stakeholders along the Cambodian rice value chain, including farmers, traders, millers, and consumers, has revealed that eating quality is a key factor of importance common to all stakeholders, especially the aroma and texture of milled rice [1]. Texture preference might depend on the culture. For instance, it has been reported that consumers from some parts of China and Vietnam favor low-amylose rice (typically with low hardness and high stickiness), while consumers from Pakistan, Philippines, Iran, and Malaysia prefer rice with an intermediate amylose content, and high-amylose rice is more popular in Sri Lanka and Myanmar [2]. An emerging challenge facing the rice industry and breeders is to modulate the texture of cooked rice for specific end-use markets.

Eating quality is a complex property, including many other attributes except for texture. Flavor is another critical factor in determining consumer choice for a particular rice variety [3,4], which is commonly evaluated by considering aroma, taste, and other sensory attributes. As an important part of rice flavor evaluation, volatile aroma compounds are an important group of aroma components having a significant role in determining rice flavor [5]. More than five hundred volatile organic components have been identified as contributing to the flavor and aroma of cooked rice [6]. For example, hexanal, 2-acetyl-1-pyrroline, and E-2-nonenal have been confirmed as key components for the aroma characteristics of cooked rice [6,7]. The content of 2-acetyl-1-pyrroline varies among rice cultivars (e.g., ~0.3 ng g^−1^ in Basmati rice) and has an odor threshold of 0.02–0.04 ng L^−1^ and 0.1 ng g^−1^ in water, which contributes to a popcorn-like aroma for rice [6,8]. Many of these flavor compounds are biologically or chemically synthesized during rice grain development, while equally important is that many others can result from the chemical breakdown during the storage of polished rice grains, such as lipid degradation by lipase. For example, brown rice had a more intense aroma than milled rice, as a higher content of lipids is located in the bran layer [6]. However, although both texture and aroma are important sensory attributes for cooked rice, there is currently limited information on the mutual relations between texture and aroma of cooked rice. It may be because cooked rice with a good texture property suffers from an inadequate flavor property. On the other hand, there were indications showing that aroma compounds can interact with amylose into complexes [9], and the texture of food is a critical factor in terms of determining the ability of amylose to form such complexes with aroma compounds [10]. In addition, the formation of amylose–aroma compound complexes could potentially inhibit the amylose–amylose intermolecular interactions during the cooling of cooked rice, which could further affect the texture of cooked rice [11,12]. Therefore, a better understanding of the relationship between the texture and aroma of cooked rice is necessary for the rice industry and breeders to develop rice or rice-based products with both desirable texture and aroma properties.

We aimed to study the relationship between the texture and aroma of cooked rice. We hypothesized that there was a causal relationship between the formation of texture and aroma for cooked rice, and a desirable texture and aroma of cooked rice could be reached through a better understanding of their relationship. To this end, we aimed to (1) conduct the investigation of the texture and aroma of five rice varieties from the Yangtze River Delta of China (a popular area for rice consumption in China) and a variety of Yueguang rice (selected due to its known superior eating quality in the world); (2) explore potential relationships between the important volatile compounds and the textural attributes of the cooked rice. China is the largest *Japonica* rice (*Oryza sativa* L. ssp. *japonica*) producer around the globe, and the Yangtze River Delta is one of the major rice-growing regions in China. However, their detailed texture and aroma profile have been rarely investigated. In this study, their textural attributes were characterized by a texture analyzer, and the volatile compound’s profile was measured with gas chromatography-ion mobility spectrometry (GC-IMS). GC-IMS combines the high separation capability of GC with the rapid responses of IMS, which has, thus, been applied in this study for the characterization of volatile organic compounds. Although sensory evaluation conducted by trained panelists is the best means to obtain consumer preferences, these tests are time-consuming, laborious, expensive, and require considerable effort and care. On the other hand, instrumental measurements are cheaper, easier, and have physiological relevance, which is invaluable for preliminary screening purposes [13]. Correlation analysis was finally performed between the textural and aroma parameters. The results could help a comprehensive understanding of the eating quality of cooked rice, which can inform the rice industry or breeders towards the assembly of desirable traits for varietal selection in future rice breeding.

## 2. Materials and Methods

Information on six different paddy rice is shown in Table 1, harvested in 2020. Yueguang (YG) rice was selected due to its known superior eating quality, including texture and aroma in the world. The other samples of rice were common rice verities, which are popular in the Yangtze River Delta of China and have proven to have superior eating quality. The rice grains were naturally dried and dehulled by a dehusker (Satake, Tokyo, Japan). A laboratory machine (Pearlest, Kett, Tokyo, Japan) was further applied for rice polishing (1 min) to obtain the white rice. Other chemicals were of analytical grade and used as received.

### 2.1. Characterization of Chemical Compositions

Total starch content was measured with the “Total starch AOAC Method 996.11/AACC Method 76-13.01” kit from Megazyme. The total crude protein content was calculated from the total nitrogen content, determined with a Kjeldahl apparatus (D5000, FoodALYT, Buchi, Essen, Germany), and a conversion factor of nitrogen to protein as 5.95 [14]. Amylose content was measured using iodine colorimetry [15]. The determination of total starch content, total crude protein content, and amylose content was performed in triplicate.

### 2.2. Appearance Characterization

The appearance of 400 rice grains was measured in triplicate, using a grain scanner (ScanMaker i800 plus, Microtek, Shanghai, China), according to a previous method with minor modifications [16].

### 2.3. Rice Cooking

Different kinds of rice were cooked according to a national standard method, ‘GB/T 15682-2008 Inspection of Grain and Oils-Method’ for rice cooking and eating quality with minor modifications [17]. Briefly, 100 g rice was rinsed two times with 300 mL of distilled water and washed with 200 mL of distilled water within 3 min. The washed rice was cooked using the pre-set cooking setting of a rice cooker (Bear DFB-B12K2, Guangdong Bear Electric Co., Ltd., Guangdong, China). A rice-to-water ratio of 1:1.3 was applied and then cooked for 40 min (cooking for 20 min and stewing for 20 min) in the rice cooker after being soaked for 30 min. Cooked rice was then cooled at room temperature for 60 min before further analysis. The rice cooking experiments were performed in triplicate.

### 2.4. Textural Analysis

Textural analysis of cooked rice was performed with a texture analyzer (TA.XT plus, Stable Micro Systems Ltd., London, UK) equipped with a P36R cylindrical probe (35 mm), following a previous method with minor modifications [18]. Test samples were collected by removing the rice material from the top 1 cm layer and adhering to the sides and bottom of the container. About 1 g of cooked rice grains was located at the center of the flat stage, and a two-cycle, force-versus-distance compression program was applied for the textural analysis. Testing parameters include a pre-test speed of 1.0 mm/s, test speed of 0.8 mm/s, post-test speed of 2.0 mm/s, 75% strain, time of 5 s, and auto-trigger force of 5 g. Texture measurements were performed five times at room temperature.

### 2.5. Analysis of Volatile Compounds by GC-IMS

Volatile compounds of cooked rice were analyzed using a GC-IMS instrument (Flavourspec^®^, G.A.S, Dortmund, Germany) according to a previous method with modifications [19]. The volatile compounds released after cooking is significantly different from those released in the field at flowering time. Briefly, a 2 g cooked rice sample was directly transferred into a 20 mL headspace vial that was subsequently incubated at 60 °C for 15 min at an agitation speed of 500 rpm (conditions that are frequently applied in the literature to balance the headspace gas [20,21]). Then, 500 μL of the headspace was automatically injected into the inlet via a heated syringe at 85 °C. The chromatographic separation was performed with a capillary column (FS-SE-54-CB-1, 15 m × 0.53 mm × 1 μm, Restek, Beijing, China). High-purity nitrogen was employed as carrier gas using the following programmed flow: 2 mL/min for 2 min and raised to 150 mL/min within 23 min. The drift tube was 98 mm long, with a drift gas flow rate of 150 mL/min. The temperature of the column and drift tube was kept at 40 °C and 45 °C, respectively. The volatile compounds were tentatively identified in the user-built database (GCxIMS Library Search 1.0.3) and NIST14 library obtained from G.A.S (Dortmund, Germany). Their quantitative comparisons were normalized to the range of 0–1000. The analytical spectrum was viewed by using the Laboratory Analytical Viewer (LAV2.2.1). The volatile compounds of different rice were tested three times.

### 2.6. Statistical Analysis

One-way ANOVA with Tukey adjustment was applied for the analysis of significant differences among different variables (*p* < 0.05) by SPSS Statistics version 19 (IBM). Correlation analysis was also analyzed by SPSS19, with *p*-value lower than 0.05 and 0.01, indicating significant and highly significant correlations, respectively.

## 3. Results and Discussion

### 3.1. Basic Rice Compositions and Rice Appearance

The total starch, protein, and amylose content for different kinds of rice are shown in Table 2. Consistent with much of the literature [12], the total starch content was about 76–81%, and the protein content was about 5–7%. In addition, these kinds of rice had an amylose content of 11% to 19%.

Rice is commonly sold and eaten as white rice, which is usually evaluated by its physical traits such as grain length, grain width, and degree of chalk [2]. Rice grain morphology, such as size and chalkiness, were, thus, characterized in the current study (Table 2). As shown in Table 2, the length of different rice grains ranged from 4.59 mm to 4.94 mm, and the width ranged from 2.76 mm to 2.85 mm. The ratio of length to width ranged from 1.66 to 1.73. WY3 rice had the largest size compared to the other samples of rice, with a length × width of 4.94 mm × 1.73 mm. WY3 rice also had the longest shape, with a ratio of length to width of 1.73. Chalk is another trait of appearance that influences consumer acceptance of rice. Chalky rice grains have opaque spots in the endosperm, which can lower the overall market value of rice, as all markets dictate the value of rice mainly depending on two traits: the proportion of broken grain and the proportion of chalk (chalk can, directly and indirectly, contribute to both traits). YG rice had the lowest, while NG5055 and YN1 rice had the highest chalky rice rate and chalkiness.

### 3.2. Textural Analysis

Textural attributes as measured by the texture analyzer for different kinds of rice are summarized in Table 3, which shows that a significant variance of textural attributes exists among different cooked rice samples. Among different attributes, hardness is commonly the dominant factor in determining the consumers’ acceptance of cooked rice, although there might be a diverse preference for rice hardness among different regions in China [12]. YG rice had a significantly higher hardness at *p* < 0.05 compared to the rest rice samples. Typically, YG rice had the highest hardness, while NG46 rice had the lowest hardness compared to the other samples of rice tested in the study. This might be related to the degree of starch retrogradation in the rice kernels, with a faster retrogradation rate contributing to a harder texture after a short period of cooling [11]. It is also supported by the lowest AC from NG46 rice, as amylose molecules have a faster retrogradation rate. On the other hand, YN1 showed the highest stickiness, while WY3 showed the lowest stickiness compared to the other samples of rice. Stickiness was shown to be largely determined by the protein and starch content and structure in the leachate [22]. It indicates that a significant difference in leached protein and starch content and structure was associated with different kinds of rice during cooking. The gumminess showed a similar trend to hardness for different cooked rice. This is expected, as gumminess can represent the energy required to chew foods into an edible form, and a harder texture indicates more energy is needed. Cohesiveness was calculated as the ratio of the peak area from the second compression divided by that from the first compression, representing the strength of internal bonds making up the cooked rice [23]. Therefore, it suggested that YG had the highest strength of internal bonds (e.g., hydrogen bonds among starch hydroxyl groups) compared to other cooked rice. Springiness can reflect the ability of cooked rice to spring back and recover its original geometry after compression [23,24]. Therefore, Table 3 suggested that different cooked rice had significantly different recovery abilities after compression.

### 3.3. Analysis of 3D Topographic Map and 2D Difference Comparison Map of the Volatile Flavor Compounds in Cooked Rice

**Figure 1:**
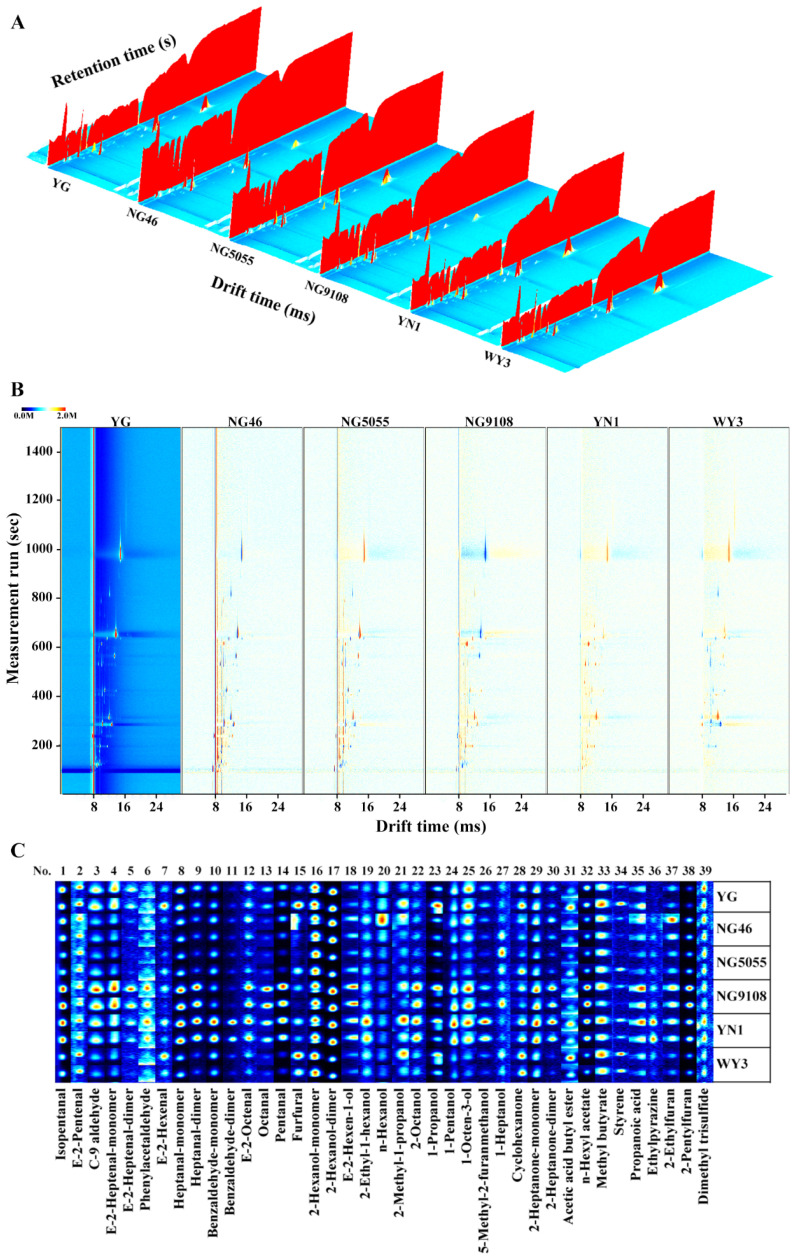
The 3D topographic map of volatile organic flavor compounds (**A**), difference comparison map of volatile organic flavor compounds (**B**), and the fingerprints of volatile organic flavor compounds (**C**) in 6 different cooked rice.

A 3D topographic map of the volatile flavor compounds found in cooked rice samples is shown in Figure 1A, in which the longitudinal, transverse, and vertical axes are gas chromatography retention time, ion migration time, and signal peak intensity after normalization, respectively. The background for the map was blue, and the red vertical line at abscissa 8.0 was the reaction ion peak (RIP, normalized). The 3D topographic map showed the original information of all volatile compounds, with each peak on the right side of the RIP peak associated with a type of volatile organic compound in the cooked rice samples. From the 3D map, the signal peak position (species) and intensity (concentration) for each volatile compound in cooked rice samples could be determined. A GC-IMS difference comparison map (Figure 1B), in which the YG spectra were selected as the reference and those of other cooked rice samples were applied as the deduction reference, was further obtained to better visualize the differences of volatile organic compounds among different cooked rice samples. The ordinate and transverse coordinates represented the gas chromatographic retention time and ion migration time, respectively. In the difference comparison map, the blue, white, and red color represent the lower, equal, and higher concentrations of volatile organic compounds in the deduction reference rice samples compared to those in the YG reference sample. Figure 1B showed that there was a large difference in the types of volatile flavor compounds among these cooked rice samples and most difference signals appeared in the drift time of 8–13 ms and retention time of 100–700 s.

### 3.4. Qualitative Results and Fingerprint Analysis of Volatile Organic Compounds in Cooked Rice Samples

The fingerprints of volatile organic compounds for all cooked rice samples are shown in Figure 1C, which were ordered according to their drift times. Each of the areas in the figure represents a characteristic volatile organic flavor compound of the sample. It shows that a total of 39 volatile organic compounds were identified in all different rice varieties. Some of these compounds showed two forms, i.e., monomers and dimers. The types and amounts of the volatile organic compounds were largely different among different cooked rice samples.

Detailed quantified information on the volatile organic compounds in all cooked rice samples are further summarized in Table 4, reordered according to their relative group abundance. Among these volatile organic compounds, 14 were aldehydes, 12 were alcohols, 3 were esters, 3 were ketones, 2 were furans, and 1 for the rest. 2-Acetyl-1-pyrroline has been detected with a higher concentration in brown rice than milled rice [25], which is not observed in the current study, possibly due to the different rice varieties applied. In addition, normal rice cooking could largely reduce the amount of 2-Acetyl-1-pyrroline [25]. Aldehydes were the most abundant compounds, which were largely produced by lipid oxidation [26]. This is consistent with the literature [27]. Aldehydes have a low sensory threshold and commonly contribute to a desirable aroma with fatty and grassy notes [28]. Among different cooked rice samples, YN1 had the highest total amount of aldehydes, followed by NG9108, YG, NG46, WY3, and NG5055. Furthermore, NG9108 had the highest amount of C-9 aldehyde, which is an aldehyde with six carbons, formed during linoleic acid oxidation and is considered a safe flavor substance in food [29]. NG9108 also had the highest amount of E-2-heptenal, which has been found in cranberries and raw potatoes as the volatile flavor compound [30]. WY3 had the highest amount of E-2-hexenal, which can be used as a flavoring agent and is also a natural compound with antibacterial activity [31]. YN1 had the highest amount of benzaldehyde, which has been shown to have an almond aroma [32]. Octanal mainly originated from the autoxidation of oleic acid [33], which was observed to be most abundant in the NG9108 sample.

On the other hand, alcohols were the second most abundant volatile organic compounds, which have been found to be largely traced from polyunsaturated fatty acids metabolism [34]. Among different cooked rice, YN1 had the highest amount, while NG5055 had the lowest amount of alcohol. 2-Pentylfuran is a product of lipid oxidation (mainly from linoleic acid) [35], which has been selected as a biomarker for aromatic rice [33]. It has also been found in this study for different cooked rice, NG9108 had the highest amount while YG had the lowest amount. The metabolic mechanisms of other volatile organic compounds in rice are poorly understood.

### 3.5. Mutual Relations between Textures and Aromas of Cooked Rice

Pearson correlation analysis was performed between the textural and aroma parameters (Table 5) to show the linear relations between textures and aromas for cooked rice. Among different textural attributes, hardness and stickiness are the most important parameters determining the consumers’ acceptance of cooked rice [22]. Hardness is positively correlated with the content of E-2-hexenal, 2-hexanol-monomer, 1-propanol, and E-2-pentenal. It has been shown that hardness is frequently determined by short-term starch retrogradation [11]. That is, a faster short-term retrogradation could form more double helices and entanglements among starch molecules during the short-term cooling process, which can contribute to a harder texture of cooked rice. For example, rice starches with relatively shorter amylose chains can have a faster short-term retrogradation rate, leading to cooked rice with a harder texture [11]. On the other hand, the lipid can form amylose–lipid complexes with amylose molecules into a single helical inclusion structure, which can inhibit the formation of double helices and entanglements between amylose and amylose as well as amylose and amylopectin molecules [36]. However, the degradation of lipids into volatile organic compounds such as E-2-hexenal, 2-hexanol-monomer, 1-propanol, and E-2-pentenal might, thus, promote the short-term starch retrogradation and, thus, the development of cooked rice with a hard texture.

On the other hand, stickiness is positively correlated with 5-methyl-2-furanmethanol and dimethyl trisulfide. Stickiness is commonly determined by the content and structural profile of leachate (e.g., starch and protein), which can form a surface layer on top of cooked rice [37]. For example, starch gelatinization in the surface layer of rice grains during parboiling may block starch leaching, consequently resulting in a less sticky texture [38]. In addition, rice stickiness is strongly correlated with leached amylopectin amount and the proportion of short amylopectin chains (degree of polymerization (DP) < 36) in the leachate [39]. The degradation of lipids into volatile organic compounds such as 5-methyl-2-furanmethanol and dimethyl trisulfide might alter the interactions among starch molecules within cooked rice kernels, which can subsequently change the structural profile of starch molecules in the leachate. This hypothesis could be tested in the future by analyzing the starch structures in the leachate of different cooked rice samples. Furthermore, as mentioned in the introduction, some aroma compounds could interact with amylose molecules into complexes [10], which could also potentially alter the structural profile of starch molecules in the leachate.

Other correlations have also been found among cohesiveness, springiness, gumminess, and volatile organic compounds. For example, gumminess has similar correlations with the volatile organic compounds with that for hardness, as both hardness and gumminess can reflect the energy required to chew foods into an edible form. Cohesiveness was positively correlated with E-2-hexenal, furfural, methyl butyrate, styrene, and E-2-pentenal, while negatively correlated with 2-ethylfuran. It suggests that the development of these volatile organic compounds can change the strength of internal bonds making up the cooked rice, such as hydrogen bonds among starch molecules. This is supported by the interactions between amylose and aroma compounds [10]. Finally, springiness was positively correlated with the amount of octanal, pentanal, 2-hexanol-dimer, E-2-hexen-1-ol, 2-heptanone-monomer, and 2-heptanone-dimer, while negatively correlated with the amount of furfural. It indicates that the development of these volatile organic compounds could affect the recovery abilities of cooked rice after compression, for example, chewing.

To sum up, the development of texture and aroma in cooked rice is a complex process, and both are important for the final eating quality of cooked rice. Considering the effects of genetic variability in commercial rice types (hybrids, Aromatics), grown environments, and cooking methodologies (e.g., steaming, high-pressure cooking, and microwave cooking involving different heating and cooling rate) on the eating quality of cooked rice, more different rice varieties grown from different locations and with different cooking conditions should be further explored in the future to obtain a holistic view on the mutual relations between the texture and aroma of cooked rice. In addition to instrumental analysis, sensory panels with different ethics should be recruited and trained to determine the eating qualities of the above-mentioned rice in the future, considering the different preferences of individual consumers for the rice quality. Nevertheless, the current pilot study, for the first time, offered many correlations between the textures and aromas. More importantly, possible mechanisms were proposed based on the scheme of effects of lipids metabolism and amylose–aroma compounds interactions on the short-term starch retrogradation and starch leaching property. The methodology of this study could be readily expanded in the future to cover the roles of different rice varieties, environmental effects, cooking conditions, and consumer preferences on the relations between the texture and aroma of cooked rice.

## 4. Conclusions

Mutual relations between the texture and aroma of cooked rice were investigated in the current study as a pilot study. There was a large difference in the textural attributes among these selected rice varieties, consistent with the literature. A total number of 39 volatile organic components were observed for these cooked kinds of rice, among which 14 were aldehydes, 12 were alcohols, 3 were esters, 3 were ketones, 2 were furans, and 1 for the rest. Many linear relations were found between the texture and aroma profile of cooked rice. For example, the hardness of cooked rice was positively correlated with the content of E-2-hexenal, 2-hexanol-monomer, 1-propanol, and E-2-pentenal, while stickiness was positively correlated with 5-methyl-2-furanmethanol and dimethyl trisulfide. Gumminess, cohesiveness, and springiness were also largely correlated with the aroma profile of cooked rice. These results could help the rice industry and breeders to develop rice products with both desirable texture and aroma properties. For example, during rice breeding and variety selection, rice seeds with appropriate contents of E-2-hexenal, 2-hexanol-monomer, 1-propanol, and E-2-pentenal could be selected to obtain cooked rice with appropriate hardness, and rice seeds with appropriate contents of 5-methyl-2-furanmethanol and dimethyl trisulfide could be selected to obtain cooked rice with appropriate stickiness.

## Figures and Tables

**Table 1:** Basic information for six kinds of *Japonica* rice applied in this study.

Samples	Sources	Location	Abbreviation
Nangeng No. 46	Jiangsu Nongken Rice Industry Group Co., Ltd.	Nantong, China	NG46
Nangeng No. 5055	Nantong Fuzhikang Rice Industry Co., Ltd.	Nantong, China	NG5055
Nangeng No. 9108	Gaoyou Tianyang rice factory	Gaoyou, China	NG9108
Yangnong No. 1	Gaoyou Younong Rice Industry Co., Ltd.	Gaoyou, China	YN1
Wuyu No. 3	Sheyang Yucheng Rice Industry Co., Ltd.	Yancheng, China	WY3
Yueguang	Japan Quannong pearl rice Co., Ltd.	Ayase, Japan	YG

**Table 2:** Basic compositions and appearance qualities for six kinds of *Japonica* rice.

Samples	Total Starch (%)	Protein (%)	AC (%)	Average Length (mm)	Average Width (mm)	Average Length/Width	Chalky Rice Rate (%)	Chalkiness (%)
NG46	78.25 ± 0.49 ^bc^	5.81 ± 0.17 ^e^	10.52 ± 0.36 ^e^	4.67 ± 0.03 ^bc^	2.78 ± 0.03 ^b^	1.69 ± 0.01 ^bcd^	12.58 ± 0.78 ^c^	3.66 ± 0.09 ^c^
NG5055	79.53 ± 0.49 ^ab^	6.76 ± 0.04 ^a^	10.64 ± 0.07 ^e^	4.64 ± 0.06 ^c^	2.78 ± 0.01 ^b^	1.67 ± 0.02 ^cd^	24.77 ± 5.32 ^ab^	9.15 ± 0.55 ^a^
NG9108	76.44 ± 1.68 ^c^	6.26 ± 0.02 ^c^	17.20 ± 0.67 ^b^	4.75 ± 0.07 ^b^	2.79 ± 0.06 ^b^	1.71 ± 0.01 ^ab^	23.81 ± 0.54 ^b^	6.72 ± 0.40 ^b^
YN1	77.07 ± 0.29 ^c^	6.45 ± 0.04 ^b^	12.20 ± 0.10 ^d^	4.59 ± 0.02 ^c^	2.77 ± 0.01 ^b^	1.66 ± 0.00 ^d^	29.70 ± 0.60 ^a^	8.88 ± 0.50 ^a^
WY3	77.85 ± 0.51 ^bc^	6.04 ± 0.01 ^d^	18.63 ± 0.09 ^a^	4.94 ± 0.05 ^a^	2.85 ± 0.01 ^a^	1.73 ± 0.01 ^a^	14.32 ± 1.86 ^c^	3.27 ± 0.37 ^c^
YG	80.97 ± 0.96 ^a^	5.32 ± 0.02 ^f^	15.61 ± 0.52 ^c^	4.65 ± 0.01 ^bc^	2.76 ± 0.00 ^b^	1.69 ± 0.00 ^bc^	10.34 ± 1.02 ^c^	2.97 ± 0.06 ^c^

Values shown are mean ± SD. Values with different letters in the same column are significantly different at *p* < 0.05.

**Table 3:** TPA characteristic parameters for six kinds of *Japonica* rice.

Samples	Hardness (g)	Stickiness (g·s)	Gumminess (g)	Cohesiveness	Springiness
NG46	10,025.26 ± 498.03 ^b^	1116.70 ± 32.70 ^b^	5092.86 ± 415.44 ^d^	0.42 ± 0.03 ^b^	0.83 ± 0.04 ^bc^
NG5055	10,261.49 ± 129.89 ^b^	770.04 ± 9.88 ^c^	5274.70 ± 129.64 ^cd^	0.44 ± 0.00 ^b^	0.83 ± 0.01 ^bc^
NG9108	10,291.83 ± 381.47 ^b^	871.16 ± 27.09 ^c^	5393.01 ± 14.92 ^bcd^	0.42 ± 0.01 ^b^	0.87 ± 0.01 ^ab^
YN1	10,959.79 ± 684.16 ^b^	1471.02 ± 88.82 ^a^	6066.69 ± 43.76 ^b^	0.44 ± 0.01 ^b^	0.85 ± 0.01 ^abc^
WY3	11,244.82 ± 659.16 ^b^	551.49 ± 10.35 ^d^	6013.26 ± 222.36 ^bc^	0.49 ± 0.01 ^a^	0.80 ± 0.03 ^c^
YG	12,852.05 ± 959.56 ^a^	1151.44 ± 140.94 ^b^	7075.90 ± 647.55 ^a^	0.49 ± 0.03 ^a^	0.83 ± 0.01 ^bc^

Values shown are mean ± SD. Values with different letters in the same column are significantly different at *p* < 0.05.

**Table 4:** Major volatile organic compounds in different cooked rice samples.

Volatiles	No.	Flavor Compounds	Drift Time (ms)	NG46	NG5055	NG9108	YN1	WY3	YG
Aldehydes	1	Isopentanal	1.2024	408.78 ± 66.44 ^b^	19.14 ± 19.14 ^c^	979.73 ± 20.27 ^a^	918.92 ± 56.31 ^a^	172.30 ± 28.15 ^c^	557.43 ± 102.48 ^b^
	2	C-9 aldehyde	1.4876	98.31 ± 14.04 ^c^	129.21 ± 106.74 ^c^	901.69 ± 98.31 ^a^	564.61 ± 58.99 ^b^	28.09 ± 28.09 ^c^	643.26 ± 47.75 ^b^
	3	E-2-heptenal-monomer	1.2515	174.85 ± 21.47 ^d^	33.74 ± 33.74 ^d^	960.12 ± 39.88 ^a^	466.26 ± 42.94 ^c^	101.23 ± 46.01 ^d^	748.47 ± 55.21 ^b^
	4	E-2-heptenal-dimer	1.6656	150.79 ± 23.81 ^c^	23.81 ± 23.81 ^c^	904.76 ± 95.24 ^a^	547.62 ± 87.30 ^b^	71.43 ± 23.81 ^c^	603.17 ± 63.49 ^b^
	5	Phenylacetaldehyde	1.259	287.88 ± 45.45 ^b^	242.42 ± 242.42 ^b^	848.48 ± 151.52 ^a^	818.18 ± 90.91 ^a^	515.15 ± 30.30 ^ab^	469.70 ± 106.06 ^ab^
	6	E-2-hexenal	1.5283	181.03 ± 43.10 ^a^	318.97 ± 181.03 ^a^	275.86 ± 275.86 ^a^	482.76 ± 206.90 ^a^	681.03 ± 232.76 ^a^	637.93 ± 362.07 ^a^
	7	Heptanal-monomer	1.3392	189.72 ± 47.87 ^c^	67.38 ± 67.38 ^c^	943.26 ± 35.46 ^a^	966.31 ± 33.69 ^a^	90.43 ± 15.96 ^c^	500.00 ± 49.65 ^b^
	8	Heptanal-dimer	1.6906	137.72 ± 35.93 ^c^	35.93 ± 35.93 ^c^	922.16 ± 77.84 ^a^	913.17 ± 86.83 ^a^	44.91 ± 2.99 ^c^	356.29 ± 38.92 ^b^
	9	Benzaldehyde-monomer	1.1491	210.46 ± 64.48 ^b^	201.95 ± 4.87 ^b^	42.58 ± 42.58 ^b^	918.49 ± 81.51 ^a^	221.41 ± 58.39 ^b^	141.12 ± 21.90 ^b^
	10	Benzaldehyde-dimer	1.4631	105.42 ± 45.18 ^b^	90.36 ± 6.02 ^b^	15.06 ± 15.06 ^b^	858.43 ± 141.57 ^a^	96.39 ± 36.14 ^b^	60.24 ± 12.05 ^b^
	11	E-2-octenal	1.3322	385.42 ± 156.25 ^bc^	62.50 ± 62.50 ^c^	864.58 ± 72.92 ^a^	895.83 ± 104.17 ^a^	114.58 ± 72.92 ^bc^	395.83 ± 20.83 ^b^
	12	Octanal	1.8172	25.42 ± 2.82 ^d^	25.42 ± 25.42 ^d^	966.10 ± 33.90 ^a^	649.72 ± 62.15 ^b^	19.77 ± 2.82 ^d^	364.41 ± 31.07 ^c^
	13	Pentanal	1.4194	253.86 ± 68.61 ^bc^	5.15 ± 5.15 ^d^	906.52 ± 93.48 ^a^	734.13 ± 73.76 ^a^	48.89 ± 9.43 ^cd^	275.30 ± 72.90 ^b^
	14	Furfural	1.0787	412.28 ± 263.16 ^a^	302.63 ± 250.00 ^a^	171.05 ± 171.05 ^a^	407.89 ± 118.42 ^a^	811.40 ± 188.60 ^a^	596.49 ± 377.19 ^a^
Alcohols	15	2-Hexanol-monomer	1.2719	230.77 ± 230.77 ^c^	543.27 ± 24.04 ^bc^	649.04 ± 43.27 ^ab^	687.50 ± 52.88 ^ab^	812.50 ± 100.96 ^ab^	951.92 ± 48.08 ^a^
	16	2-Hexanol-dimer	1.5623	685.04 ± 14.62 ^b^	140.04 ± 53.43 ^c^	968.50 ± 31.50 ^a^	867.83 ± 21.93 ^a^	21.93 ± 21.93 ^c^	718.79 ± 70.87 ^b^
	17	E-2-Hexen-1-ol	1.1772	346.85 ± 58.56 ^c^	193.69 ± 94.59 ^cd^	986.49 ± 13.51 ^a^	684.68 ± 27.03 ^b^	13.51 ± 13.51 ^e^	45.05 ± 45.05 ^de^
	18	2-Ethyl-1-hexanol	1.4217	208.96 ± 119.40 ^b^	141.79 ± 22.39 ^b^	253.73 ± 14.93 ^b^	880.60 ± 119.40 ^a^	246.27 ± 67.16 ^b^	29.85 ± 29.85 ^b^
	19	n-Hexanol	1.324	637.17 ± 362.83 ^a^	39.82 ± 39.82 ^b^	35.40 ± 26.55 ^b^	101.77 ± 48.67 ^ab^	66.37 ± 39.82 ^ab^	349.56 ± 39.82 ^ab^
	20	2-Methyl-1-propanol	1.1705	355.42 ± 307.23 ^a^	48.19 ± 48.19 ^a^	307.23 ± 259.04 ^a^	728.92 ± 271.08 ^a^	548.19 ± 367.47 ^a^	391.57 ± 343.37 ^a^
	21	2-Octanol	1.4319	90.00 ± 30.00 ^b^	60.00 ± 60.00 ^b^	940.00 ± 60.00 ^a^	990.00 ± 10.00 ^a^	140.00 ± 0.00 ^b^	80.00 ± 0.00 ^b^
	22	1-Propanol	1.1149	259.33 ± 68.07 ^a^	149.28 ± 149.28 ^a^	461.41 ± 378.29 ^a^	511.87 ± 187.87 ^a^	557.46 ± 189.36 ^a^	703.35 ± 296.65 ^a^
	23	1-Pentanol	1.2483	350.81 ± 76.61 ^b^	48.39 ± 24.19 ^c^	733.87 ± 40.32 ^a^	891.13 ± 108.87 ^a^	32.26 ± 32.26 ^c^	403.23 ± 48.39 ^b^
	24	1-Octen-3-ol	1.1547	620.48 ± 114.46 ^b^	138.55 ± 30.12 ^c^	945.78 ± 54.22 ^a^	873.49 ± 90.36 ^a^	30.12 ± 30.12 ^c^	831.33 ± 0.00 ^ab^
	25	5-Methyl-2-Furanmethanol	1.5638	128.87 ± 56.70 ^bc^	25.77 ± 25.77 ^c^	324.74 ± 67.01 ^b^	855.67 ± 144.33 ^a^	72.16 ± 51.55 ^bc^	314.43 ± 5.15 ^b^
	26	1-Heptanol	1.3877	300.00 ± 250.00 ^a^	762.50 ± 237.50 ^a^	600.00 ± 325.00 ^a^	12.50 ± 12.50 ^a^	400.00 ± 175.00 ^a^	50.00 ± 50.00 ^a^
Esters	27	Acetic acid butyl ester	1.2352	419.35 ± 64.52 ^a^	104.84 ± 104.84 ^a^	419.35 ± 32.26 ^a^	580.65 ± 161.29 ^a^	620.97 ± 379.03 ^a^	572.58 ± 266.13 ^a^
	28	n-Hexyl acetate	1.412	51.17 ± 13.16 ^d^	48.25 ± 48.25 ^d^	998.54 ± 1.46 ^a^	783.63 ± 38.01 ^b^	39.47 ± 7.31 ^d^	540.94 ± 58.48 ^c^
	29	Methyl butyrate	1.4401	400.00 ± 88.89 ^a^	400.00 ± 0.00 ^a^	344.44 ± 344.44 ^a^	555.56 ± 200.00 ^a^	722.22 ± 277.78 ^a^	677.78 ± 322.22 ^a^
Ketones	30	Cyclohexanone	1.1486	250.00 ± 107.14 ^a^	386.90 ± 375.00 ^a^	250.00 ± 250.00 ^a^	767.86 ± 113.10 ^a^	934.52 ± 65.48 ^a^	636.90 ± 220.24 ^a^
	31	2-Heptanone-monomer	1.2596	303.03 ± 90.91 ^bc^	49.24 ± 34.09 ^cd^	875.00 ± 49.24 ^a^	928.03 ± 71.97 ^a^	45.45 ± 45.45 ^d^	382.58 ± 109.85 ^b^
	32	2-Heptanone-dimer	1.6248	402.3 ± 149.43 ^b^	109.2 ± 5.75 ^bc^	827.59 ± 114.94 ^a^	885.06 ± 114.94 ^a^	5.75 ± 5.75 ^c^	304.60 ± 74.71 ^bc^
Furans	33	2-Ethylfuran	1.2989	762.82 ± 237.18 ^a^	173.08 ± 6.41 ^bc^	641.03 ± 89.74 ^a^	551.28 ± 51.28 ^ab^	0.00 ± 0.00 ^c^	96.15 ± 44.87 ^c^
	34	2-Pentylfuran	1.2512	373.35 ± 33.57 ^c^	282.30 ± 38.15 ^cd^	948.63 ± 51.37 ^a^	690.23 ± 17.80 ^b^	197.86 ± 5.60 ^d^	13.73 ± 13.73 ^e^
Terpenes	35	Styrene	1.5024	126.87 ± 126.87 ^a^	328.36 ± 328.36 ^a^	179.10 ± 134.33 ^a^	470.15 ± 97.01 ^a^	895.52 ± 104.48 ^a^	634.33 ± 335.82 ^a^
Alkenes	36	E-2-Pentenal	1.1036	281.25 ± 31.25 ^a^	250.00 ± 62.50 ^a^	156.25 ± 156.25 ^a^	531.25 ± 343.75 ^a^	625.00 ± 312.50 ^a^	906.25 ± 31.25 ^a^
Pyrazines	37	Ethyl pyrazine	1.1253	353.45 ± 25.86 ^bc^	275.86 ± 17.24 ^c^	431.03 ± 17.24 ^b^	948.28 ± 51.72 ^a^	224.14 ± 86.21 ^c^	8.62 ± 8.62 ^d^
Acids	38	Propanoic acid	1.2678	357.14 ± 166.67 ^ab^	7.94 ± 7.94 ^b^	809.52 ± 190.48 ^a^	738.10 ± 182.54 ^a^	190.48 ± 111.11 ^b^	412.70 ± 126.98 ^ab^
Others	39	Dimethyl trisulfide	1.3047	638.89 ± 138.89 ^abc^	416.67 ± 27.78 ^bcd^	250.00 ± 83.33 ^cd^	777.78 ± 0.00 ^ab^	138.89 ± 138.89 ^d^	833.33 ± 166.67 ^a^

Values shown are mean ± SD. Values with different letters in the same row are significantly different at *p* < 0.05.

**Table 5:** Pearson correlation analysis between textural parameters and aromas for cooked rice samples.

		Hardness	Stickiness	Gumminess	Cohesiveness	Springiness
Aldehydes	Isopentanal	0.058	0.62	0.166	−0.336	0.764
	C-9 aldehyde	0.262	0.418	0.326	−0.16	0.81
	E-2-heptenal-monomer	0.338	0.334	0.376	−0.052	0.708
	E-2-heptenal-dimer	0.241	0.415	0.307	−0.16	0.775
	Phenylacetaldehyde	0.072	0.312	0.201	−0.136	0.545
	E-2-hexenal	0.825 *	−0.121	0.845 *	0.930 **	−0.555
	Heptanal-monomer	0.081	0.605	0.203	−0.313	0.792
	Heptanal-dimer	−0.015	0.562	0.111	−0.382	0.806
	Benzaldehyde-monomer	0	0.677	0.144	−0.135	0.103
	Benzaldehyde-dimer	−0.003	0.714	0.148	−0.179	0.203
	E-2-octenal	−0.077	0.654	0.045	−0.458	0.795
	Octanal	0.024	0.385	0.122	−0.326	0.830 *
	Pentanal	−0.138	0.505	−0.027	−0.485	0.832 *
	Furfural	0.597	−0.257	0.569	0.873 *	−0.889 *
Alcohols	2-Hexanol-monomer	0.838 *	−0.085	0.864 *	0.788	−0.156
	2-Hexanol-dimer	0.025	0.737	0.094	−0.449	0.824 *
	E-2-Hexen-1-ol	−0.488	0.348	−0.389	−0.747	0.866 *
	2-Ethyl-1-hexanol	−0.192	0.592	−0.031	−0.315	0.273
	n-Hexanol	0.046	0.37	−0.025	−0.077	−0.124
	2-Methyl-1-propanol	0.312	0.467	0.431	0.257	−0.134
	2-Octanol	−0.218	0.432	−0.076	−0.466	0.719
	1-Propanol	0.838 *	0.194	0.877 *	0.695	−0.096
	1-Pentanol	−0.016	0.755	0.108	−0.434	0.787
	1-Octen-3-ol	0.166	0.749	0.233	−0.329	0.796
	5-Methyl-2-Furanmethanol	0.185	0.817 *	0.335	−0.167	0.506
	1-Heptanol	−0.619	−0.744	−0.685	−0.357	0.058
Esters	Acetic acid butyl ester	0.572	0.269	0.633	0.528	−0.245
	n-Hexyl acetate	0.152	0.475	0.252	−0.239	0.808
	Methyl butyrate	0.804	−0.054	0.813 *	0.932 **	−0.681
Ketones	Cyclohexanone	0.58	−0.035	0.642	0.755	−0.607
	2-Heptanone-monomer	−0.055	0.657	0.069	−0.447	0.815 *
	2-Heptanone-dimer	−0.197	0.683	−0.076	−0.586	0.848 *
Furans	2-Ethylfuran	−0.619	0.512	−0.567	−0.861 *	0.66
	2-Pentylfuran	−0.583	0.221	−0.48	−0.747	0.765
Terpenes	Styrene	0.684	−0.291	0.693	0.918 **	−0.719
Alkenes	E-2-Pentenal	0.952 **	0.204	0.950 **	0.887 *	−0.46
Pyrazines	Ethyl pyrazine	−0.414	0.575	−0.261	−0.56	0.442
Acids	Propanoic acid	0.004	0.574	0.114	−0.354	0.735
Others	Dimethyl trisulfide	0.419	0.888 *	0.448	0.009	0.246

* is significant at *p* < 0.05, while ** is significant at *p* < 0.01.

## Data Availability

The datasets generated for this study are available on request to the corresponding author.

## References

[1] Concepcion J.C.T., Ouk M., Zhao D., Fitzgerald M.A. (2015). The need for new tools and investment to improve the accuracy of selecting for grain quality in rice. Field Crops Res..

[2] Calingacion M., Laborte A., Nelson A., Resurreccion A., Concepcion J.C., Daygon V.D., Mumm R., Reinke R., Dipti S., Bassinello P.Z. (2014). Diversity of global rice markets and the science required for consumer-targeted rice breeding. PLoS ONE.

[3] Biao Y., Chanjuan Z., Ming Y., Dechun H., McClements D.J., Zhigang H., Chongjiang C. (2019). Influence of gene regulation on rice quality: Impact of storage temperature and humidity on flavor profile. Food Chem..

[4] Verma D.K., Srivastav P.P. (2022). Extraction, identification and quantification methods of rice aroma compounds with emphasis on 2-Acetyl-1-Pyrroline (2-AP) and its relationship with rice quality: A comprehensive review. Food Rev. Int..

[5] Verma D.K., Srivastav P.P. (2020). A paradigm of volatile aroma compounds in rice and their product with extraction and identification methods: A comprehensive review. Food Res. Int..

[6] Hu X., Lu L., Guo Z., Zhu Z. (2020). Volatile compounds, affecting factors and evaluation methods for rice aroma: A review. Trends Food Sci. Technol..

[7] Bergman C.J., Delgado J.T., Bryant R., Grimm C., Cadwallader K.R., Webb B.D. (2000). Rapid gas chromatographic technique for quantifying 2-acetyl-1-pyrroline and hexanal in rice (*Oryza sativa* L.). Cereal Chem..

[8] Mathure S.V., Wakte K.V., Jawali N., Nadaf A.B. (2011). Quantification of 2-Acetyl-1-pyrroline and other rice aroma volatiles among indian scented rice cultivars by HS-SPME/GC-FID. Food Anal. Methods.

[9] Ma R., Tian Y., Chen L., Cai C., Jin Z. (2019). Effects of cooling rate on retrograded nucleation of different rice starch-aromatic molecule complexes. Food Chem..

[10] Arvisenet G., Le Bail P., Voilley A., Cayot N. (2002). Influence of physicochemical interactions between amylose and aroma compounds on the retention of aroma in food-like matrices. J. Agric. Food Chem..

[11] Li C., Luo J., Zhang C., Yu W. (2020). Causal relations among starch chain-length distributions, short-term retrogradation and cooked rice texture. Food Hydrocolloid.

[12] Tao K., Yu W., Prakash S., Gilbert R.G. (2019). High-amylose rice: Starch molecular structural features controlling cooked rice texture and preference. Carbohydr. Polym..

[13] Tao K., Yu W., Prakash S., Gilbert R.G. (2020). Investigating cooked rice textural properties by instrumental measurements. Food Sci. Hum. Wellness.

[14] Li C., Cao P., Wu P., Yu W., Gilbert R.G., Li E. (2021). Effects of endogenous proteins on rice digestion during small intestine (in vitro) digestion. Food Chem..

[15] Wang Z., Hu Z., Deng B., Gilbert R.G., Sullivan M.A. (2022). The effect of high-amylose resistant starch on the glycogen structure of diabetic mice. Int. J. Biol. Macromol..

[16] Okpala N.E., Potcho M.P., An T., Ahator S.D., Duan L., Tang X. (2020). Low temperature increased the biosynthesis of 2-AP, cooked rice elongation percentage and amylose content percentage in rice. J. Cereal Sci..

[17] Zhao Q., Xi J., Xu D., Jin Y., Wu F., Tong Q., Yin Y., Xu X. (2022). A comparative HS-SPME/GC-MS-based metabolomics approach for discriminating selected rice varieties from different regions of China in raw and cooked form. Food Chem..

[18] Zhao Y., Henry R.J., Gilbert R.G. (2021). Starch structure-property relations in Australian wild rices compared to domesticated rices. Carbohydr. Polym..

[19] Yang Y., Qian M.C., Deng Y., Yuan H., Jiang Y. (2022). Insight into aroma dynamic changes during the whole manufacturing process of chestnut-like aroma green tea by combining GC-E-Nose, GC-IMS, and GC x GC-TOFMS. Food Chem..

[20] Zhang X., Dai Z., Fan X., Liu M., Ma J., Shang W., Liu J., Strappe P., Blanchard C., Zhou Z. (2020). A study on volatile metabolites screening by HS-SPME-GC-MS and HS-GC-IMS for discrimination and characterization of white and yellowed rice. Cereal Chem..

[21] Liu J., Liu M., Liu Y., Jia M., Wang S., Kang X., Sun H., Strappe P., Zhou Z. (2020). Moisture content is a key factor responsible for inducing rice yellowing. J. Cereal Sci..

[22] Li H., Gilbert R.G. (2018). Starch molecular structure: The basis for an improved understanding of cooked rice texture. Carbohydr. Polym..

[23] Szczesniak A.S. (1962). Classification of textural characteristics. J. Food Sci..

[24] Sahi S.S., Little K., Ananingsih V.K. (2014). “Quality Control” Bakery Products Science and Technology.

[25] Buttery R.G., Ling L.C., Juliano B.O., Turnbaugh J.G. (1983). Cooked rice aroma and 2-acetyl-1-pyrroline. J. Agric. Food Chem..

[26] García-Llatas G., Lagarda M.J., Clemente G., Farré R. (2006). Monitoring of headspace volatiles in milk-cereal-based liquid infant foods during storage. Eur. J. Lipid Sci. Technol..

[27] Sun Z., Lyu Q., Chen L., Zhuang K., Wang G., Ding W., Wang Y., Chen X. (2022). An HS-GC-IMS analysis of volatile flavor compounds in brown rice flour and brown rice noodles produced using different methods. LWT—Food Sci. Technol..

[28] Zhang M., Li L., Song G., Wang H., Wang H., Shen Q. (2020). Analysis of volatile compound change in tuna oil during storage using a laser irradiation based HS-SPME-GC/MS. LWT—Food Sci. Technol..

[29] Lehtonen M., Kekalainen S., Nikkila I., Kilpelainen P., Tenkanen M., Mikkonen K.S. (2020). Active food packaging through controlled in situ production and release of hexanal. Food Chem. X.

[30] Gaona Colman E., Blanco M.B., Barnes I., Wiesen P., Teruel M.A. (2017). Mechanism and Product Distribution of the O3-Initiated Degradation of (E)-2-Heptenal, (E)-2-Octenal, and (E)-2-Nonenal. J. Phys. Chem. A.

[31] Germinara G.S., Conte A., De Cristofaro A., Lecce L., Di Palma A., Rotundo G., Del Nobile M.A. (2012). Electrophysiological and behavioral activity of (E)-2-hexenal in the granary weevil and its application in food packaging. J. Food Prot..

[32] Yu H., Xie T., Xie J., Chen C., Ai L., Tian H. (2020). Aroma perceptual interactions of benzaldehyde, furfural, and vanillin and their effects on the descriptor intensities of Huangjiu. Food Res. Int..

[33] Griglione A., Liberto E., Cordero C., Bressanello D., Cagliero C., Rubiolo P., Bicchi C., Sgorbini B. (2015). High-quality Italian rice cultivars: Chemical indices of ageing and aroma quality. Food Chem..

[34] Ferreira V., López R., Cacho J.F. (2000). Quantitative determination of the odorants of young red wines from different grape varieties. J. Sci. Food Agric..

[35] Resconi V.C., Bueno M., Escudero A., Magalhaes D., Ferreira V., Campo M.M. (2018). Ageing and retail display time in raw beef odour according to the degree of lipid oxidation. Food Chem..

[36] Wang S., Chao C., Cai J., Niu B., Copeland L., Wang S. (2020). Starch–lipid and starch–lipid–protein complexes: A comprehensive review. Compr. Rev. Food Sci. Food Saf..

[37] Li C., Li E., Gong B. (2022). Main starch molecular structures controlling the textural attributes of cooked instant rice. Food Hydrocolloid.

[38] Li H., Yan S., Yang L., Xu M., Ji J., Mao H., Song Y., Wang J., Sun B. (2021). Starch gelatinization in the surface layer of rice grains is crucial in reducing the stickiness of parboiled rice. Food Chem..

[39] Li H., Yang J., Yan S., Lei N., Wang J., Sun B. (2019). Molecular causes for the increased stickiness of cooked non-glutinous rice by enzymatic hydrolysis of the grain surface protein. Carbohydr. Polym..

